# Beetroot Plus Vitamin C for Performance and Recovery: Protocol of a Double‐Blind, Placebo‐Controlled, Randomized Crossover Trial in Semi‐Professional Wrestlers

**DOI:** 10.1002/hsr2.72218

**Published:** 2026-05-03

**Authors:** Maedeh Nojoumi, Alireza Hosseini Kakhki, Mohammad Ali Sardar, Ali Jafari, Ali Jafarzadeh Esfehani, Reza Rezvani

**Affiliations:** ^1^ Department of Nutrition, Faculty of Medicine Mashhad University of Medical Sciences Mashhad Iran; ^2^ Department of Sports Physiology, Faculty of Sports Sciences Ferdowsi University of Mashhad Mashhad Iran; ^3^ Department of General Education, Faculty Medicine Mashhad University of Medical Sciences Mashhad Iran; ^4^ Department of Community Nutrition, Faculty of Nutrition Sciences and Food Technology Shahid Beheshti University of Medical Sciences Tehran Iran

**Keywords:** beet, BS, CK, LDH, nitrate, sport, supplement, Wingate

## Abstract

**Introduction:**

In competitive sports, minor performance enhancements can significantly impact outcomes, driving athletes to utilize nutritional supplements, though many lack robust scientific evidence. Inorganic nitrate (NO₃⁻) stands out as a well‐supported ergogenic aid, particularly for high‐intensity activities, enhancing both performance metrics and psychological factors like perceived exertion. Wrestling, characterized by short, intense bursts of activity, necessitates efficient energy metabolism and recovery strategies. This protocol describes a trial to evaluate the acute effects of vitamin C‐enriched beetroot supplementation on the performance of semi‐professional wrestlers, assessed through upper and lower body Wingate tests.

**Method:**

This study will conduct a double‐blind, randomized, placebo‐controlled crossover trial involving 28 semi‐professional wrestlers. Participants will be assigned to receive either a beetroot juice containing 8.4 mmol of nitrate and 90 mg of vitamin C or a placebo. Randomization will be facilitated through a web‐based tool, ensuring blinding with placebo drinks. The primary outcome will be maximal anaerobic power, while secondary outcomes will include mean anaerobic power, fatigue index, time to exhaustion, and metabolic markers associated with muscle damage, such as creatine kinase and lactate dehydrogenase. The trial is registered at IRCT20240407061440N1.

**Results:**

Not applicable (study protocol).

**Conclusion:**

This protocol describes a rigorous trial to evaluate potential ergogenic and recovery benefits of vitamin C‐enriched beetroot in wrestling‐specific anaerobic performance, addressing gaps in multi‐muscle group testing.

## Abstract (highlights)

**Strengths and limitations of this study:**

- This protocol employs a rigorous double‐blind, randomized, placebo‐controlled crossover design, minimizing bias and enhancing internal validity for assessing acute supplementation effects in semi‐professional wrestlers.
- The study replicates wrestling‐specific demands by alternating upper and lower body Wingate tests, providing novel insights into multi‐muscle group performance and recovery.
- Comprehensive controls for dietary and lifestyle confounders, including nitrate‐rich food restrictions and standardized diets, strengthen the reliability of findings on ergogenic aids.
- Limitations include the focus on acute effects only, without chronic supplementation assessment, and the sample restricted to male semi‐professional wrestlers aged 18–24, potentially limiting generalizability to other populations or sports.
- The crossover design assumes adequate washout, but individual variability in nitrate metabolism could introduce minor carryover effects despite the 14‐day period.

**Summary:**

- While existing studies have demonstrated the ergogenic effects of beetroot supplementation in combat sports, including its potential to improve performance and recovery, limited research has specifically examined its combined use with vitamin C in wrestling, particularly in relation to upper‐ and lower‐body anaerobic performance and muscle damage markers in semi‐professional athletes. This highlights the need for targeted research into the effects of beetroot supplements on multi‐muscle group activities. The current study's findings could inform nutritional strategies for combat sports and influence dietary guidelines within sports organizations, thereby promoting evidence‐based recommendations for supplementation in athletics.

**Abbreviations**
CK: creatine kinase
CONSORT: consolidated standards of reporting trials
LDH: lactate dehydrogenase
NO: nitric oxide
NO₃⁻: inorganic nitrate
RPE: ratings of perceived exertion
SPIRIT: standard protocol items: recommendations for interventional trials
WAnT: wingate anaerobic test

## 1 Introduction

In competitive sports, even marginal enhancements in performance can profoundly influence competition outcomes, prompting athletes to increasingly rely on nutritional supplements. However, many such supplements lack robust scientific validation for their efficacy. To bridge this evidence gap, the International Olympic Committee has developed a categorization system for nutritional supplements, evaluating them based on the strength of supporting research for athletic performance benefits [1].

Among these, inorganic nitrate (NO₃⁻) has emerged as a promising ergogenic aid, particularly for high‐intensity sports, with evidence from meta‐analyzes demonstrating improvements in aerobic and anaerobic performance among recreationally active individuals [2, 3]. Recent studies, further confirm that beetroot‐derived nitrate supplementation enhances muscle endurance, time to exhaustion, and power output during high‐intensity efforts, while also reducing oxygen consumption and perceived exertion [4, 5, 6, 7, 8, 9, 10, 11]. These benefits extend to reductions in systemic blood pressure and improved efficiency in submaximal exercise.

Beyond physiological effects on skeletal muscle, psychological factors such as mood and ratings of perceived exertion (RPE) play a critical role in exercise performance [12]. Excessive use of ergogenic supplements can elevate subjective tension, a state of internal readiness for challenging tasks [13, 14]. Consequently, consuming nitrate‐rich sources like beetroot or beetroot juice prior to competition may lead significant performance gains [15], especially in anaerobic‐dominant activities.

Ascorbic acid (vitamin C) in beetroot juice augments nitric oxide (NO) bioavailability by promoting the reduction of nitrate to nitrite [16, 17], and acts as a redox‐active cofactor [16, 17, 18], modulates redox‐sensitive signaling pathways [19], enhances endothelial function [20], and may influence mitochondrial and metabolic efficiency during high‐intensity exercise. In combination with polyphenols, it optimizes nitrate conversion to NO while limiting unwanted nitrogen oxide byproducts [18]. Recent evidence suggests potential synergy between vitamin C and nitrate, enhancing urinary nitrate excretion and possibly supporting recovery, though data on acute combined effects in anaerobic performance remain limited [21]. However, the evidence for combined nitrate + vitamin C supplementation in high‐intensity anaerobic exercise remains limited and mixed. While some studies indicate improved NO bioavailability and reduced oxidative stress with acute co‐ingestion, broader reviews on antioxidant supplementation (including high‐dose vitamin C) during intense exercise show inconsistent or neutral effects on performance and recovery, potentially due to interference with adaptive signaling pathways [22, 23].

Wrestling exemplifies a sport reliant on anaerobic energy pathways, with high‐intensity bursts lasting 6 to 60 s primarily driven by glycolytic metabolism [24]. While acute beetroot supplementation has been extensively studied for its impact on upper and lower body Wingate Anaerobic Test (WAnT) outcomes, gaps persist in examining repeated, alternating upper‐ and lower‐body WAnT performances, a protocol that better mimics wrestling's demands for multi‐muscle group engagement [25]. Prior research has focused on consecutive efforts within the same body region, overlooking the explosive, sequential involvement of both upper and lower body typical in combat sports.

Furthermore, evidence on beetroot's role in attenuating muscle damage is mixed. A 2024 study showed beetroot consumption reduces creatine kinase (CK) and lactate dehydrogenase (LDH) levels, offering protection against damage [26]. with similar findings in sports like fencing [27]. However, these benefits were absent in soccer or marathon running [28, 29], likely due to variations in training status, exercise modality, supplementation protocols, and intermittent demands. Given these inconsistencies and promising results in high‐intensity contexts, there is a clear need for a controlled interventional study that replicates wrestling‐specific conditions, emphasizing dual‐muscle group involvement.

### 1.1 Objectives

The primary objective is to evaluate the acute effects of vitamin C‐enriched beetroot supplementation on maximal anaerobic power during upper and lower body Wingate tests in semi‐professional wrestlers.

Secondary objectives include assessing impacts on mean anaerobic power, fatigue index, time to peak power, and muscle damage markers (creatine kinase, lactate dehydrogenase, blood glucose), as well as ratings of perceived exertion.

### 1.2 Hypotheses

We hypothesize that acute supplementation with vitamin C‐enriched beetroot juice (8.4 mmol nitrate and 90 mg vitamin C) may enhance anaerobic performance metrics and reduce muscle damage compared to placebo, potentially driven by increased nitric oxide production and antioxidant effects. These hypotheses will be tested in the proposed trial.

## 2 Methods and Analysis

**Figure 1:**
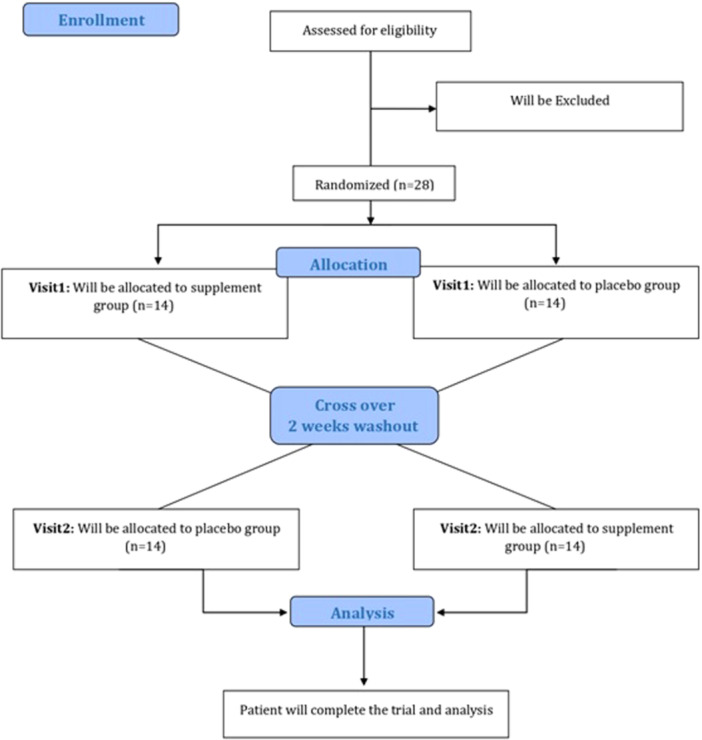
CONSORT 2010 Flow Diagram for participant enrollment, allocation, follow‐up, and analysis in this crossover trial.

The protocol for the present double‐blind, randomized, placebo‐controlled, crossover study is meticulously aligned with the Standard Protocol Items: Recommendations for Interventional Trials (SPIRIT) guidelines [30] for trial protocols and the Guidelines for reporting of statistics for clinical research in urology [31] (Figure 1). Ethical approval has been obtained from the Medical Ethics Committee of Mashhad University of Medical Sciences (IR.MUMS.MEDICAL.REC.1403.011), and the study has been officially registered with the Iranian Registry of Clinical Trials (IRCT20240407061440N1) with a registration date of June 7, 2024.

### 2.1 Protocol Version and Trial Status

This protocol is version 1.0, dated 1 August 2025. Recruitment has not yet commenced and is planned to begin in September 2025, with completion expected by December 2025.

### 2.2 Study Design and Setting

This double‐blind, crossover study will involve 28 semi‐professional wrestlers, who will receive both vitamin C‐enriched beetroot juice and placebo in random order, separated by a 14‐day washout period. Participants will be selected by their respective wrestling coaches and will be randomly assigned to receive either a 240 mL dose of a vitamin C‐enriched beetroot juice or a placebo. The beetroot juice will contain 8.4 mmol of nitrate derived from beetroot powder produced through a freeze‐drying process, along with natural mineral nitrate (3.2%), phenolic compounds, ascorbic acid, carotenoids, and betalains. Additionally, the beetroot juice for the intervention group will include 90 mg of vitamin C, with no additives or preservatives. The 90 mg dose of vitamin C was chosen based on pharmacokinetic data showing that intakes in the 75–100 mg range achieve near‐maximal plasma saturation in healthy adults [32], thereby supporting nitrate‐related nitric oxide processes without entering high‐dose pharmacological ranges that could potentially interfere with adaptive signaling.

The following inclusion criteria will be adopted:

- Healthy semi‐professional wrestlers aged 18–24 years.
- Interested in participating in the project and following the study protocol.
- Having more than 2 years of wrestling experience.
- Not being an elite athlete.
- Not performing any type of physical exercise 72 h before each session.
- The exclusion criteria will be as follows:
- Smoking or consuming alcohol.
- Consuming caffeine‐containing drinks 24 h before the start of the intervention.
- Having a history of cardiovascular, lung, metabolic, neurologic, or orthopedic disorders that could limit cycle ergometry performance.
- Utilizing nitrate‐containing pharmaceuticals, such as nitroglycerin or isosorbide.
- Taking nutritional supplements in the 6 months prior to the study onset.
- Using mouthwash or oral antiseptic within the past 4 weeks.
- Taking any herbal medicine.
- Having an allergy to red beets.

Participants will be required to abstain from caffeine for 24 h (per exclusion criteria), ensure at least 7 h of sleep, and avoid cognitively demanding tasks for 12 h before each session to control potential confounders. The application of oral antiseptics has the potential to inhibit elevated blood NO₂⁻ concentrations following the consumption of NO₃⁻ due to their bactericidal properties targeting oral bacteria. Consequently, participants will be instructed to avoid brushing their teeth, using mouthwash, chewing gum, or consuming sweets that may contain bactericidal agents like chlorhexidine or xylitol for a period of 24 h before the testing sessions.

**Figure 2:**
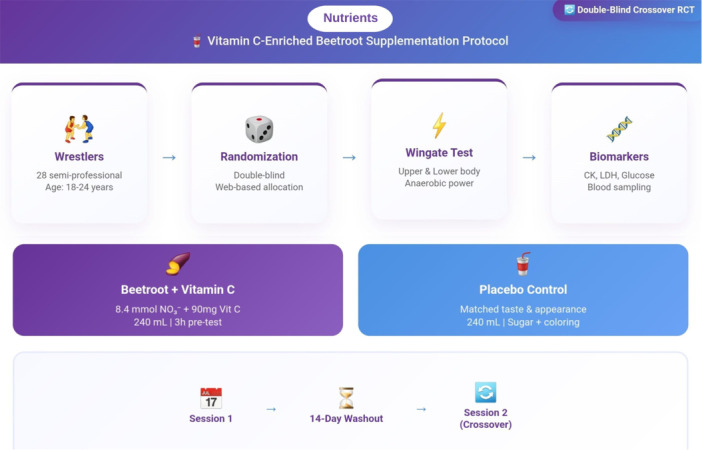
Overview of the double‐blind, randomized, placebo‐controlled crossover trial design, including intervention, washout, and outcome assessments.

Participants will be instructed to maintain a fixed diet of 60% carbohydrate, 30% fat, and 10% protein, avoiding high‐fat foods for 48 h prior to each test session. In addition, they will be given a list of NO₃⁻‐rich vegetables (including beets, celery, arugula, lettuce, spinach, turnips, endives, chives, parsley, and cabbage) to avoid the day before the study begins. A 72‐h food recall will be conducted to ensure adherence to a standardized diet, minimizing dietary confounders. Supplements will be administered 3 h before Wingate tests to optimize nitrate [33], vitamin C [32], and polyphenol bioavailability [34], aligning with peak plasma concentrations. The restriction on the intake of NO₃⁻‐rich foods 48 h prior to each session will ensure that participants in the placebo group are sufficiently depleted of NO₃⁻ (Figure 2).

### 2.3 Participant Timeline

**Figure 3:**
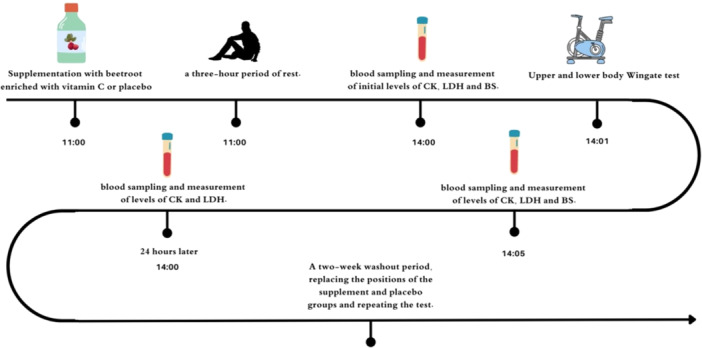
Detailed participant timeline showing timing of supplementation, blood sampling and Wingate tests (upper then lower body) across the two sessions separated by 14‐day washout.

Participants will attend two laboratory visits, separated by a 14‐day washout period. Each visit will follow this schedule:

- T = 0 h: Arrival, confirmation of adherence to pre‐test instructions, supplement administration (beetroot or placebo).
- T = 3 h: Baseline blood sample collection, anthropometric measurements.
- T = 3+ hours: Upper body Wingate test, followed by lower body Wingate test (1–4 min later), with RPE assessment during tests.
- Immediate post‐test: Blood sample collection.
- T = 27 h (24 h post‐test): Follow‐up blood sample (Figure 3).

### 2.4 Intervention Group

Participants in the intervention group will be administered a single sports drink containing a blend of red beets and vitamin C (8.4 mmol of nitrate and 90 mg of vitamin C). After a 3‐h interval, baseline blood samples will be collected from the subjects. The Wingate upper body functional test will be conducted immediately thereafter, followed by the Wingate lower body test, which will occur 1 to 4 min later. Blood samples will be obtained immediately following the Wingate lower body test and again 24 h later. Additionally, participants will complete the RPE questionnaire during both testing sessions.

### 2.5 Control Group

Participants assigned to the control group will receive a beverage that visually resembles the beetroot supplement. The placebo drink will consist of a neutral solution containing 0.4 mg/mL citric acid, 0.2 mg/mL food coloring, and 0.25 g/mL sugar, formulated to match the appearance, taste, and texture of the beetroot juice without containing nitrates, vitamin C, or polyphenols.

The beetroot juice intervention will be similarly supplemented with 0.25 g/mL sugar to ensure identical sweetness and caloric content, maintaining effective blinding. Both drinks (240 mL each) will be prepared by a third‐party supplier to ensure consistency and double‐blinding.

After a 3‐h interval, a baseline blood sample will be collected from the subjects. The Wingate upper body functional test will be performed immediately thereafter, followed by the Wingate lower body test, which will be conducted 1 to 4 min later. Blood samples will be collected immediately after the Wingate lower body test and again 24 h later. Additionally, participants will complete the RPE questionnaire during both test sessions.

Given that the half‐life of nitrate is approximately 5 to 8 h, a 14‐day washout period is considered sufficient to effectively reduce any residual effects that may arise from prior nitrate supplementation [35]. Since participants will serve as their own controls, sessions will be scheduled at the same time each day to maintain consistency.

The study's design is illustrated in Figure 3. Throughout the experimental phase, each participant will complete two visits to the laboratory.

Studies have also demonstrated that the performance of high‐intensity lower body exercises is not significantly compromised by prior upper body activity. As such, it will be feasible to conduct lower body testing and training either preceding or following upper body sessions without negatively affecting the power output of the lower body [36]. In this study, we will administer the upper body Wingate test, followed by the lower body Wingate test. This sequential testing design will enable a thorough assessment of the effects of the two supplements on performance across different muscle groups.

### 2.6 Primary Outcomes

The principal outcome measure for this study will be the anaerobic maximum power attained during the two‐stage Wingate test.

### 2.7 Secondary Outcomes

Secondary outcome measures will include anaerobic mean power, anaerobic minimum power, time to peak power, fatigue index, and various indicators to evaluate muscle damage, including levels of creatine kinase, lactate dehydrogenase, and blood glucose.

### 2.8 Power Calculation and Sample Size Estimate

Sample size was calculated based on mean power data from a previous study by Williams et al. (2020) [37] (placebo: 508.14 ± 117.55 W; BRJ: 607.36 ± 112.28 W). The required sample size was calculated to be 22 participants for a crossover design considering 95% confidence and 80% power using the following equation:

$$n=\frac{(k\times \delta_{1}^{2}+\delta_{2}^{2}){\left(Z_{1-\alpha /2}+Z_{1-\beta }\right)}^{2}}{{∆}^{2}}.$$

In the above equation, n is the sample size in each group, k is the ratio of the groups (which is 1 in this study), δ is the standard deviation in each group, Δ is the difference of group means, Z_{1‐α/2} is considered 1.96 for a 95% confidence interval, and Z_{1‐β} is the power of the study. To account for potential participant dropout and ensure completion of all study sessions, 28 participants were recruited.

### 2.9 Recruitment

Participant recruitment will be conducted at the Faculty of Sports Sciences at Ferdowsi University of Mashhad in Mashhad, Iran. The selection of participants will adhere to specified inclusion and exclusion criteria.

### 2.10 Eligibility

Individuals expressing interest in participation will undergo an assessment to determine their eligibility based on the established criteria. Eligible participants will be provided with a consent form to complete.

### 2.11 Consent Process

Informed consent will be obtained by the principal investigator or trained staff during the initial screening visit. Participants will receive verbal and written explanations of the study, risks, and benefits, with time for questions. Consent forms will be signed in duplicate, with one copy provided to the participant.

### 2.12 Confidentiality

Participant data will be anonymized using unique codes. Personal information will be stored separately from study data and accessible only to the research team. All procedures will comply with the Declaration of Helsinki and local privacy laws.

### 2.13 Allocation: Sequence Generation, Concealment, and Implementation

This double‐blind, placebo‐controlled, crossover study will randomize 28 semi‐professional wrestlers to receive either beetroot juice enriched with 90 mg vitamin C (8.4 mmol nitrate) or a placebo drink (240 mL each) in random order, with a 14‐day washout period to eliminate carryover effects. Randomization will be performed using a web‐based tool, with allocation details concealed in consecutively numbered opaque envelopes managed by an independent staff member.

Blinding will be maintained for participants, assessors (care providers), and investigators. The intervention and placebo drinks will be visually identical. Drinks will be prepared and labeled with participant numbers by the independent staff member, ensuring that participants and assessors (conducting Wingate tests, blood sampling, and RPE questionnaires) remain unaware of group allocations. Investigators overseeing the study will have no access to allocation details until data collection is completed. Data will be coded with participant numbers to maintain unbiased analysis.

#### 2.13.1 Sequence Generation

Randomization will be performed using a web‐based tool to generate a balanced crossover sequence (1:1 allocation to start with beetroot or placebo).

#### 2.13.2 Concealment Mechanism

Allocation details will be concealed in consecutively numbered opaque envelopes prepared by an independent staff member not involved in assessments.

#### 2.13.3 Implementation

The independent staff member will generate the sequence, enroll participants after eligibility screening, and assign interventions by providing the pre‐labeled drinks.

### 2.14 Safety

Although the occurrence of complications and adverse events linked to the intervention is considered improbable, participants will be prompted to self‐report any symptoms or adverse events they experience. The sole previously recorded side effects are beeturia (the appearance of red urine) and red stools [7, 38]. To ensure participant safety, the facilitator and a graduate student will be readily accessible by phone, allowing participants to promptly report any issues or inquiries. Any potential complications will be documented and addressed in consultation with a physician. Furthermore, communication and participant monitoring will be enhanced through the use of social networking platforms such as Telegram and WhatsApp.

### 2.15 Data Monitoring, Harms, and Auditing

Given the low‐risk nature of this nutritional intervention trial, no formal data monitoring committee will be established. Interim analyzes will not be conducted, as the study duration is short. Potential adverse events (e.g., gastrointestinal discomfort, beeturia) will be monitored via participant self‐reports during visits and follow‐up calls. Serious adverse events, though unlikely, will be reported immediately to the ethics committee and documented in trial records. Independent audits of trial conduct will be performed by the university's research office at midpoint and completion to ensure protocol adherence.

### 2.16 Study Assessments

Participants will undergo initial and concluding evaluations encompassing several assessments. These will include a comprehensive questionnaire gathering general information, the International Physical Activity Questionnaire, a 72‐h food recall prior to testing, and administration of the Rate of Perceived Exertion (RPE) Scale during upper and lower body Wingate tests using the Borg scale [39]. Additionally, participants will receive a detailed list of foods rich in nitrates and will be instructed to abstain from consuming these items throughout the study duration.

### 2.17 Data Collection Methods

Data will be collected using standardized forms and electronic records. Wingate test data will be recorded directly from the Monark ergometer software. Blood samples will be analyzed in a certified laboratory using automated analyzers for CK, LDH, and glucose. RPE will be assessed via the Borg scale immediately post‐test. A 72‐h food recall will be administered by trained staff to verify dietary adherence. All data will be double‐entered to minimize errors.

### 2.18 Anthropometric Measurements

Height will be measured on a flat surface without shoes using a stadiometer. Weight, fat mass (FM), and fat‐free mass (FFM) will be assessed using a bioelectrical impedance analyzer (InBody 720 Body Composition Analyzer).

### 2.19 Device Base Parameters

To evaluate upper body Wingate test indices, a Monark cycle ergometer (Ergomedic 891E, Vansbro, Sweden) will be used, and to evaluate lower body Wingate test indices, a Monark cycle ergometer (Ergomedic 891E, Vansbro, Sweden) will be utilized. The load (kgf) will be set at 7.5% of each participant's body weight for the lower body Wingate test and 5% of body weight for the upper body Wingate test [40].

### 2.20 Laboratory Analysis

To assess serum levels of certain parameters, 10 mL venous blood samples will be collected at each blood draw, and the levels of lactate dehydrogenase, creatine kinase, and blood sugar will be measured.

### 2.21 Statistical Analysis

Data will be analyzed using SPSS v26. Normality will be assessed via the Shapiro‐Wilk test. Comparison of the within‐subject effects of BRJ (90 mg vitamin C) versus placebo will be evaluated using paired t‐tests (for normal data) or Wilcoxon signed‐rank tests (for non‐normal data). A univariate General Linear Model (GLM) will be used to assess treatment, sequence, and treatment‐sequence (carryover) effects. Statistical significance will be set at *p* < 0.05.

### 2.22 Data Management

Data will be stored securely on password‐protected servers at Mashhad University of Medical Sciences, with access limited to authorized investigators. Participant identifiers will be coded to ensure confidentiality. Quality checks will include range validation and consistency audits. Data backups will occur daily, and the database will comply with local data protection regulations.

### 2.23 Patient and Public Involvement

Patients and the public were not involved in the conceptualization, execution, reporting, or dissemination of our research findings.

### 2.24 Protocol Amendments

Any amendments to the protocol will be submitted for approval to the Medical Ethics Committee of Mashhad University of Medical Sciences and updated in the trial registry (IRCT20240407061440N1). Changes will be communicated to participants via updated consent forms if necessary, and documented in publications.

## 3 Discussion

Nitrates have gained popularity due to their perceived potential as an ergogenic aid. Beetroot constitutes a robust antioxidant containing nitrates that is associated with improvements in exercise performance [41, 42].

High‐intensity exercise is more effective than endurance activities in raising blood nitric oxide (NO) levels, a response that can be enhanced by beetroot juice (BJ) supplementation. During anaerobic exercise, there is a marked decrease in oxygen partial pressure and pH levels within the muscle and blood [43], conditions that promote the conversion of nitrate (NO₃⁻) to nitric oxide (NO) [18]. Evidence suggests that the nitrate (NO₃⁻) pathway is more active in hypoxic and acidic conditions associated with intense physical activity [44], prompting research into the performance‐enhancing effects of beet juice supplementation [45, 46, 47, 48, 49, 50, 51].

On the other hand, the concurrent consumption of these biomolecules alongside ascorbic acid may promote the synthesis of nitric oxide (NO) by enhancing the reduction of nitrate (NO₃⁻) and/or nitrite (NO₂⁻) within the oral cavity or gastrointestinal tract [16, 17, 52]. Chronic or high‐dose antioxidant supplementation has been associated with mixed outcomes, sometimes blunting exercise‐induced adaptations by reducing reactive oxygen species signaling [19].

The 30‐second maximum sprint test performed on a cycle ergometer, known as the Wingate test, is an effective method for assessing performance in high‐intensity exercise by quantifying power output and glycolytic capacity [53]. The application of beetroot juice has been shown to enhance mean power output and speed, expedite the attainment of absolute power, and mitigate or sustain fatigue index levels in participants undergoing the Wingate maximal effort test [48].

However, findings on the effectiveness of beetroot juice for improving performance and increasing power during high‐intensity efforts still remain inconsistent across studies [54]. Knowledge about the effects of beetroot supplementation on recovery after ultra‐endurance sports also remains insufficient. Therefore, this study aims to examine the influence of vitamin C‐enriched beetroot supplementation and provide evidence on its potential effects during endurance exercise on Wingate Anaerobic Test functional results and mitigating muscle damage. It should be noted that creatine kinase (CK) and lactate dehydrogenase (LDH) are indirect markers of muscle damage and tissue stress, reflecting only one dimension of post‐exercise recovery [55, 56]. These biomarkers do not fully capture neuromuscular, perceptual, or metabolic aspects of recovery [57].

### 3.1 Limitations

A key limitation of this protocol is the absence of a beetroot‐only supplementation arm (without added vitamin C). This design prevents isolation of the specific contribution of vitamin C to any observed effects, limiting mechanistic interpretation regarding whether performance/recovery benefits stem predominantly from nitrate pathways or from antioxidant synergy. Future studies should consider a three‐arm design (placebo, beetroot alone, beetroot + vitamin C) to address this gap. Additionally, while the crossover design strengthens within‐subject comparisons, the relatively small sample (*n* = 28) may limit detection of subtle effects on secondary biochemical markers. Another limitation is the absence of baseline biochemical assessment of vitamin C status or broader redox markers, which could contribute to inter‐individual variability in responses to nitrate and vitamin C. While the 72‐h dietary recall offers descriptive insight into habitual vitamin C intake, it does not replace direct measurement. This aspect represents an opportunity for future studies with targeted biochemical profiling. Furthermore, the study is limited to male semi‐professional wrestlers aged 18–24 years; therefore, findings may not generalize to female athletes, older individuals, elite‐level competitors, or those in aerobic‐dominant sports, where differences in training status, hormonal profiles, and redox responses could influence outcomes.

## Author Contributions

R.R. and M.N. conceptualized the study design. A.H.K., M.A.S., A.J., and M.N. will oversee the study implementation and participant communication. A.J.E. will conduct the data analysis. The manuscript was developed by M.N. and A.J., with revisions provided by R.R., A.J., and A.H.K. All authors have thoroughly reviewed and approved the final version of the manuscript. Reza Rezvani had full access to all of the data in this study and takes complete responsibility for the integrity of the data and the accuracy of the data analysis.

## Ethics Statements

As this is a protocol article, the informed consent of participants is not applicable at this stage. However, all participants involved in the subsequent phases of the study will be required to provide written informed consent for publication of their data and findings. Ethical approval has been obtained from the Medical Ethics Committee of Mashhad University of Medical Sciences (IR.MUMS.MEDICAL.REC.1403.011).

## Consent

Patients and/or the public were not involved in the design, execution, reporting, or dissemination plans of this research.

## Conflicts of Interest

The authors declare no conflicts of interest.

## Transparency Statement

The lead author Reza Rezvani affirms that this manuscript is an honest, accurate, and transparent account of the study being reported; that no important aspects of the study have been omitted; and that any discrepancies from the study as planned (and, if relevant, registered) have been explained.

## Data Availability

No further information is accessible at this protocol stage. Upon study completion, de‐identified participant data may be made available upon reasonable request to the corresponding author, subject to ethical approvals.
